# The disparities in prognostic prediction and annualized hazard function in different molecular subtypes between young Chinese and White American women with breast cancer

**DOI:** 10.3389/fonc.2023.1199492

**Published:** 2023-07-10

**Authors:** Yuanyuan Zeng, Jun Wang, Xiaorong Zhong, Zhongzheng Xiang, Tian Yang, Siting Yu, Zelei Dai, Ningyue Xu, Lei Liu

**Affiliations:** ^1^ Division of Head & Neck Tumor Multimodality Treatment, Cancer Center, and State Key Laboratory of Biotherapy, West China Hospital, Sichuan University, Chengdu, China; ^2^ Department of Radiation Oncology, Cancer Center, West China Hospital, Sichuan University, Chengdu, China; ^3^ Breast Center, Cancer Center, West China Hospital, Sichuan University, Chengdu, China

**Keywords:** breast cancer, molecular subtype, young women, race/ethnicity, prognosis

## Abstract

**Background and objectives:**

The prognostic disparities in different molecular subtypes between young Chinese and White American breast cancer patients remain unclear. The goal of this study was to explore the prognostic differences in different molecular subtypes between Chinese and White American patients aged ≤ 40 years.

**Methods:**

We included Chinese and White female breast cancer patients at or under the age of 40 from the Surveillance, Epidemiology, and End Results database (SEER) and the West China Hospital of Sichuan University. The chi-square test, log-rank test, and Cox proportional hazards model were employed to evaluate the distribution and survival disparities in the two racial/ethnic cohorts and different molecular subtypes. An annualized hazard function was used to calculate the annual failure rate among different molecular subtypes.

**Results:**

This study included 20,859 female breast cancer patients at or under the age of 40, of whom 18,400 were White women and 2,459 were Chinese women. With a median follow-up time of 47 months, the 5-year breast cancer-specific survival (BCSS) rates for young Chinese and White women were 93.9% and 90.0%, respectively (*P*< 0.001). Molecular subtype was found to be a significant predictor in both young Chinese and White patients (*P*< 0.001), but different trends were observed in the two racial/ethnic cohorts when exploring the association between BCSS and molecular subtypes. Among young White patients, the hormone receptor (HoR) (+)/epidermal growth factor receptor 2 (HER2) (+) subtype had the best 5-year BCSS rate, while in young Chinese patients, the HoR (+)/HER2 (+) and HoR (+)/HER2 (-) showed comparable survival curves and both showed superior 5-year BCSS than other subtypes. Stratification by molecular subtypes, young Chinese patients demonstrated a superior 5-year BCSS in HoR (+)/HER2 (-) (96.3% vs 92.9%, *P*< 0.001) and triple-negative subtypes (88% vs 81.7%, *P*= 0.006) compared to young White American patients, while no significant differences were found in HoR (+)/HER2 (+) and HER2 enriched tumors. The annual hazard function for BCSS showed that there were significantly different trends in the HoR (+)/HER2 (-) and HoR (+)/HER2 (+) subtypes between young Chinese and White patients.

**Conclusions:**

There are disparities in prognosis and annualized hazard function between young Chinese and White females with breast cancer in different molecular subtypes.

## Introduction

Breast cancer is the most prevalent female malignant tumor and the second leading cause of death for women worldwide (1). Approximately 7-10% of women with breast cancer patients are under the age of 40 (2). However, young females with breast cancer have always been regarded as a unique group with distinct biological and clinical characteristics (3). Previous studies revealed that young females with breast cancer had an increased probability to develop high grade, high proliferation, and more aggressive tumors (4, 5), as well as worse survival and outcomes than older women (5-year relative survival rate: 86% vs 91%) (6).

As the comprehension of tumor biology has advanced, growing evidence suggests that younger breast cancer patients’ inferior survival outcomes may be associated with their propensity for different molecular subtypes (7, 8). The incidence of breast cancer, as well as its mortality and survival rates, differ significantly based on the molecular subtype (9, 10). Luminal A tumors exhibit the highest occurrence rate but the lowest mortality rates among women, while luminal B tumors exhibit higher tumor proliferation than luminal A tumors (11, 12). Epidermal growth factor receptor 2 (HER2) enriched and triple-negative tumors, on the other hand, exhibit a correlation with inferior survival outcomes despite being less common (13, 14). Most tumors in all age cohorts are hormone receptor (HoR) positive and HER2-negative, although young women are more likely than older women to develop invasive subtypes of tumors with poor prognostic characteristics, such as triple-negative and HER2 enriched tumors (15, 16).

Additionally, racial/ethnic disparities exist in breast carcinogenesis and the prognosis of young women with breast cancer (16–19). Compared to the United States, China has a lower incidence of breast cancer, but the incidence has been rising in young Chinese women, leading to a higher percentage of Chinese patients aged 40 or younger (20). Furthermore, several studies have indicated that young Asians have a greater propensity for developing advanced stages, lower rates of poorly differentiated tumors and invasive molecular subtypes, but better breast cancer survival in comparison to young White women (19, 21, 22).

Age, molecular subtype, and race/ethnicity all have a substantial impact on breast cancer survival outcomes (8, 23). Young breast cancer patients exhibit more aggressive subtypes and unfavorable prognostic features (24, 25). Nevertheless, the prognostic disparities in molecular subtypes among young breast cancer patients of diverse race/ethnicity backgrounds remain unclear. A few research have examined the disparities of race/ethnicity in survival when considering modern tumor subtypes among young women (8, 26, 27). However, due to inadequate inclusion of women from Asian or other ethnic groups, or focusing solely on a specific subtype, such as triple-negative tumors, these studies have not comprehensively described disparities in survival across races/ethnicities in various subtypes of breast cancer (8, 26, 27). Given the rising incidence of breast cancer among young Chinese women, it is essential to explore prognostic differences in subtypes between young Chinese women and other ethnicities. Using combined data from the Surveillance, Epidemiology, and End Results (SEER) database and the West china hospital of Sichuan university, the study accessed the differences in prognostic prediction and annualized hazard function in different molecular subtypes between young Chinese and White American breast cancer patients, to develop a greater understanding of the biological characteristics of breast cancer in young individuals of various races/ethnicities and to provide a scientific basis for precise personalized treatment.

## Methods

### Data source and study population

The patient information was retrieved from the SEER database and West china hospital of Sichuan university. The SEER database, which collects comprehensive information on cancer incidence, treatments, and clinical outcomes from cancer registries, covers approximately one-third of the United States population but only includes a very small number of Chinese breast cancer patients. For better comparisons with Chinese women, most data on Chinese women were retrieved from the Breast Cancer Information Management System (BCIMS) database at West China Hospital of Sichuan University, which prospectively gathers comprehensive patient information from medical records, including demographic statistics, tumor features, treatment specifics, and follow-up information. Given that the status of HER2 amplification in the SEER database was not recorded until 2010, we extracted newly diagnosed White American or Chinese breast cancer patients at or under the age of 40 from the SEER database between 2010 and 2018 in our study. And Chinese breast cancer patients at or under the age of 40 diagnosed between 2008 and 2019 from the BCIMS database were included in our study.

In addition, due to the unavailability of Ki67 expression status in the SEER database, molecular subtypes of patients from this database cannot be accurately distinguished between Luminal A and Luminal B. Consistent with other studies based on the SEER database (19, 28, 29), this study categorized the molecular subtypes into the following four subtypes based on the progesterone receptor (PR)/estrogen receptor (ER) (ER and PR were combined into HoR) and HER2 status: HoR (+)/HER2 (-), HoR (+)/HER2 (+), HER2 enriched (HoR (-)/HER2 (+)) and triple-negative (HoR (-)/HER2 (-)).

In the SEER database, race and ethnicity are reported in five mutually exclusive categories: Non-Hispanic White (White), Non-Hispanic Black (Black), Non-Hispanic Asian/Pacific Islander (Asian and Pacific Islander), Non-Hispanic American Indian/Alaska Native (American Indian/Alaska Natives) and Hispanic. And Asians were subclassified as Chinese, Japanese, Korean, Filipino, Vietnamese, South Asian, Southeast Asian, or other Asian. In our study, only Chinese and White Americans from the SEER database and Chinese individuals from West China Hospital (BCIMS database) were included. We selected White (code 01) and Chinese (code 04) based on “Race/ethnicity” codes in the SEER database for analysis. To maintain consistency, the Race/ethnicity included in this study was classified into two categories: White American (henceforth referred to as White) and Chinese (including Chinese from SEER and West China Hospital).

Patients were recruited for this study based on the following criteria: (1) histologically confirmed to have invasive breast cancer; (2) Chinese or White American women; (3) age at diagnosis ≤ 40 years old; and (4) detailed record on tumor differentiation, the status of ER, PR, HER2, molecular subtype, tumor stage, nodal stage, clinical stage (AJCC staging system 7^th^ edition), surgery, chemotherapy, and radiotherapy administration. Exclusion criteria included: (1) male patients with breast cancer; (2) diagnosed with distant metastasis or contralateral breast cancer; and (3) coexisted of one or more cancers.

### Variables and endpoints

The clinicopathological and demographic characteristics were collected as followed: race/ethnicity (White American and Chinese), tumor grade (well differentiated, moderately differentiated, poorly differentiated/undifferentiated, unknown), ER status, PR status, HER2 status, molecular subtype (HoR (+)/HER2 (-), HoR (+)/HER2 (+), HER2 enriched and triple-negative), tumor stage (T0, T1, T2, T3, T4), nodal stage (N0, N1, N2, N3), clinical stage (I, II, III), surgery, chemotherapy, and radiotherapy. This study’s endpoint was breast cancer-specific survival (BCSS), which was measured as the time between the diagnosis of breast cancer and the occurrence of breast cancer-related death. The duration of survival was calculated from the time of breast cancer diagnosis until the time of death or the last follow-up.

### Statistical analysis

The disparities in clinicopathological characteristics between young Chinese and White American females with breast cancer were examined using the chi-square test. BCSS in the different cohorts was analyzed by Kaplan–Meier, and the log-rank test estimated differences in survival. The life-table method was used to calculate the survival rates. Multivariate analysis of significant factors was conducted by the Cox proportional hazards model. The maximum likelihood estimate of a piecewise exponential model was performed to calculate the annualized hazard rates for different racial/ethnic groups, representing the percentage of events occurring within a specified time interval. A two-sided p value of < 0.05 was deemed statistically significant. All analyses were conducted using IBM SPSS Statistics (IBMCorp., Armonk, N.Y., USA; version 26.0).

## Results

### Patient demographics and tumor characteristics

This study finally included 20,859 female breast cancer patients aged ≤ 40 years. We identified 18,938 eligible patients (including 18,400 White women and 538 Chinese women) from the SEER database and 1,921 Chinese women from the BCIMS database according to the inclusion criteria. Of them, 18,400 (88.2%) were White individuals and 2,459 (11.8%) were Chinese. Among all the patients, most patients had poorly differentiated or undifferentiated tumor grade (n= 9,904, 47.5%) and T2 tumor size (n= 9,258, 44.4%), and approximately 47.0% of them had regional lymph node metastasis. Notably, more than half of the eligible patients had ER positive (n= 15,281, 73.3%), PR positive (n= 13,268, 65.3%), HER2 negative (n= 15,639, 75.0%), or HoR (+)/HER2 (-) subtype (n= 11,897, 57%) breast cancer.

In terms of the distribution of pathology and clinical characteristics between White American and Chinese individuals, differences in several variables were found to be significant (*P*< 0.001). Compared with White women, Chinese women demonstrated a higher tendency to exhibit positive PR status, HER2 amplification, and HER2 enriched subtype. Tumor grade with poorly differentiated or undifferentiated was more prevalent in White women than in Chinese women patients. Fewer T1 and T3 but more T2 and T4 tumors were observed in Chinese women. More low nodal stages were observed in Chinese patients than in White patients. Moreover, the clinical stage showed more stage I and II tumors in White women, whereas there were more stage III tumors in Chinese patients. Regarding treatment, surgery was administered to over 95% of patients across all racial/ethnic groups, and 48% of White participants and 46.4% of Chinese participants received radiotherapy. However, there were disparities in the use of chemotherapy, and a significantly greater proportion of Chinese women received chemotherapy (*P*< 0.001). After stratification by molecular subtype, it can be observed that Chinese women received chemotherapy at a higher rate than White American women across all molecular subtypes (**Table S1**). **Table 1** listed detailed tumor characteristics according to race and ethnicity.

**Table 1:** Participants’ characteristics.

Characteristic	Total n= 20,859 (%)	White American n= 18,400 (%)	Chinese n= 2,459 (%)	*P* value^a^
**Grade**				< 0.001
Well differentiated	1,634 (7.8)	1,544 (8.4)	90 (3.7)	
Moderately differentiated	6,868 (32.9)	6,109 (33.2)	759 (30.9)	
Poorly differentiated or undifferentiated	9,904 (47.5)	8,900 (48.4)	1,004 (40.8)	
Unknown	2,453 (11.8)	1,847 (10.0)	606 (24.6)	
**ER status**				0.299
Positive	15,281 (73.3)	13,501 (73.4)	1,780 (72.4)	
Negative	5,578 (26.7)	4,899 (26.6)	679 (27.6)	
**PR status**				< 0.001
Positive	13,628 (65.3)	11,935 (64.9)	1,693 (68.8)	
Negative	7,231 (34.7)	6,465 (35.1)	766 (31.2)	
**HER2 status**				< 0.001
Positive	5,220 (25.0)	4,534 (24.6)	686 (27.9)	
Negative	15,639 (75.0)	13,866 (75.4)	1,773 (72.1)	
**Molecular subtype**				< 0.001
HoR(+)/HER-2(-)	11,897 (57)	10,508 (57.1)	1,389 (56.5)	
HoR(+)/HER-2(+)	3,785 (18.1)	3,325 (18.1)	460 (18.7)	
HER2 enriched	1,435 (6.9)	1,209 (6.6)	226 (9.2)	
Triple-negative	3,742 (17.9)	3,358 (18.3)	384 (15.6)	
**Tumor stage**				< 0.001
T0	14 (0.1)	13 (0.0)	1 (0.0)	
T1	8,684 (41.6)	7,759 (42.2)	925 (37.6)	
T2	9,258 (44.4)	8,043 (43.7)	1,215 (49.4)	
T3	2,155 (10.3)	1,983 (10.8)	172 (7.0)	
T4	748 (3.6)	602 (3.3)	146 (6.0)	
**Nodal stage**				
N0	11,060 (53.0)	9,882 (53.7)	1,178 (47.9)	< 0.001
N1	7,134 (34.2)	6,350 (34.5)	784 (31.9)	
N2	1,636 (7.9)	1,378 (7.5)	258 (10.5)	
N3	1,029 (4.9)	790 (4.3)	239 (9.7)	
**Clinical stage**				< 0.001
1	6,540 (31.4)	5,906 (32.1)	634 (25.8)	
2	10,228 (49.0)	9,027 (49.1)	1,201 (48.8)	
3	4,091 (19.6)	3,467 (18.8)	624 (25.4)	
**Surgery**				0.123
Yes	20,018 (96.0)	17,644 (95.9)	2,374 (96.5)	
No	841 (4.0)	756 (4.1)	85 (3.5)	
**Chemotherapy**				< 0.001
Yes	16,478 (79.0)	14,280 (77.6)	2,198 (89.4)	
No	4,381 (21.0)	4,120 (22.4)	261 (10.6)	
**Radiotherapy**				0.135
Yes	9,965 (47.8)	8,825 (48.0)	1,140 (46.4)	
No	10,894 (52.2)	9,575 (52.0)	1,319 (53.6)	

HoR, hormone receptor; HER2, epidermal growth factor receptor 2.

a P value from chi-square test.

### Survival and prognosis analysis for the entire cohort

**Figure 1:**
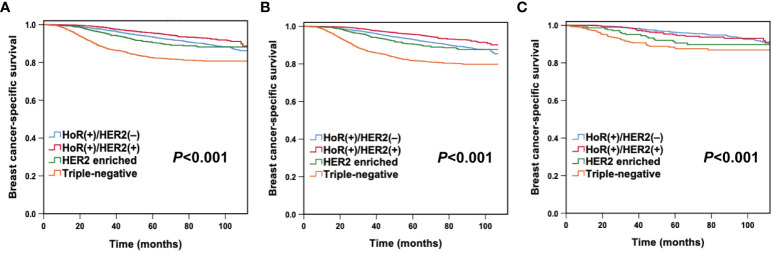
Breast cancer-specific survival by molecular subtypes in the entire cohort **(A)**, young White women **(B)**, and young Chinese women **(C)**.

With a median follow-up period of 47 months (ranging from 0 to 173 months), there were 1,617 deaths and 1,406 breast cancer-specific deaths in the entire cohort. The 5-year OS and BCSS were 90.5% and 91.7%, respectively. In this study, young White women had lower 5-year OS (90.0% vs 93.9%, *P*< 0.001) and BCSS (91.2% vs 94.2%, *P*< 0.001) than young Chinese women. When estimated across different subtypes of the whole cohort, Kaplan–Meier curves showed that patients with the triple-negative subtype had the lowest 5-year BCSS rates (HoR (+)/HER2 (-) vs. HoR (+)/HER2 (+) vs. HER2 enriched vs. triple-negative: 93.4% vs. 95.5% vs. 90.4% vs. 82.5%, *P*< 0.001; **Figure 1A**). Multivariate Cox analysis indicated that both race/ethnicity and molecular subtype had significant prognostic predictive value in the entire cohort (**Table 2**). Young Chinese patients were 47% less likely to die of breast cancer than young White patients (95% CI, 0.434-0.647; *P*< 0.001), while the risk of BCSS for the patients with the triple-negative subtype was 1.904 times higher than that for HoR (+)/HER2 (-) (95% CI, 1.677-2.162; *P*< 0.001) (**Table 2**).

**Table 2:** Multivariate analysis for breast cancer-specific survival in the whole cohort.

Variables	Multivariate analysis	
	HR (95% CI)	*P* value^a^
Race and ethnicity		
White women	1 (reference)	N/A^b^
Chinese women	0.530 (0.434-0.647)	< 0.001
Grade		
Well differentiated	1 (reference)	N/A
Moderately differentiated	1.704 (1.180-2.462)	0.005
Poorly differentiated or undifferentiated	2.958 (2.057-4.254)	< 0.001
Unknown	2.544 (1.569-4.126)	< 0.001
Molecular subtype		
HoR(+)/HER-2(-)	1 (reference)	N/A
HoR(+)/HER-2(+)	0.534 (0.446-0.641)	< 0.001
HER2 enriched	0.801(0.647-0.992)	0.042
Triple-negative	1.904 (1.677-2.162)	< 0.001
Clinical stage		
1	1 (reference)	N/A
2	2.673 (2.183-3.273)	< 0.001
3	9.011 (7.340-11.063)	< 0.001
Surgery		
Yes	1 (reference)	N/A
No	0.401 (0.330-0.487)	< 0.001
Chemotherapy		
Yes	1 (reference)	N/A
No	1.019 (0.842-1.234)	0.848
Radiotherapy		
Yes	1 (reference)	N/A
No	1.013 (0.901-1.138)	0.831

HR, hazard ratio; CI, confidence interval; HoR, hormone receptor; HER2, epidermal growth factor receptor 2.

a P value: Adjusted for all relevant factors using multivariate Cox proportional hazards models.

b N/A, Not applicable.

### Survival and prognosis analysis according to race/ethnicity

After stratification by race/ethnicity, we examined the association between molecular subtypes and BCSS. Kaplan–Meier curves (**Figures 1B, C**) and multivariate analysis (**Table 3** **)** suggested that the molecular subtype of breast cancer served as a substantial prognostic factor in both young Chinese and White patients. Among young White patients, the 5-year BCSS varied by molecular subtype. Specifically, young White patients with HoR (+)/HER2 (+) subtype had the best 5-year BCSS rate, whereas those with triple-negative breast cancer had the lowest 5-year BCSS rate (HoR (+)/HER2 (-) vs. HoR (+)/HER2 (+) vs. HER2 enriched vs. triple-negative: 92.9% vs. 95.6% vs. 90.4% vs. 81.6%, *P*< 0.001; **Figure 1B**). The hazard ratios (HRs) for BCSS when comparing different molecular subtypes in young White women are presented in **Table 3**. We observed that the HR of BCSS was highest in triple-negative breast cancer (HR,1.879; 95% confidence interval (CI), 1.644-2.149; *P*< 0.001) and lowest in HoR (+)/HER2 (+) breast cancer (HR, 0.495; 95% CI, 0.407-0.604; *P*< 0.001) among young White women. Nevertheless, the results observed among young Chinese patients were slightly different. Similar to young White women, young Chinese individuals with triple-negative tumors showed the lowest 5-year BCSS, but the HoR (+)/HER2 (+) and HoR (+)/HER2 (-) subtypes demonstrated comparable survival curves, and both showed superior 5-year BCSS across all subtypes (HoR (+)/HER2 (-) vs. HoR (+)/HER2 (+) vs. HER2 enriched vs. triple-negative: 96.3% vs. 95.0% vs. 90.5% and 88.0%, *P*< 0.001; **Figure 1C**). Moreover, compared to HoR (+)/HER2 (-), young Chinese patients with triple-negative breast cancer had 2.022 times higher HRs for BCSS (95% CI, 1.355-3.016; *P*< 0.001), while no significant discrepancies were found among those patients with other two molecular subtypes (**Table 3**).

**Table 3:** HRs for breast cancer-specific survival of molecular subtype according to race/ethnicity.

Variables	White women		Chinese women	
	HR (95% CI)	*P* value^a^	HR (95% CI)	*P* value^a^
Molecular subtype				
HoR(+)/HER-2(-)	1 (reference)	N/A	1 (reference)	N/A
HoR(+)/HER-2(+)	0.495 (0.407-0.604)	< 0.001	0.830 (0.519-1.327)	0.436
HER2 enriched	0.750 (0.594-0.948)	0.016	1.157 (0.682-1.961)	0.589
Triple-negative	1.879 (1.644-2.149)	< 0.001	2.022 (1.355-3.016)	0.001

HR, hazard ratio; CI, confidence interval; HoR, hormone receptor; HER2, epidermal growth factor receptor 2.

a Adjusted for tumor grade, clinical stage, surgery, chemotherapy, and radiotherapy.

### Survival and prognosis analysis according to molecular subtypes

**Figure 2:**
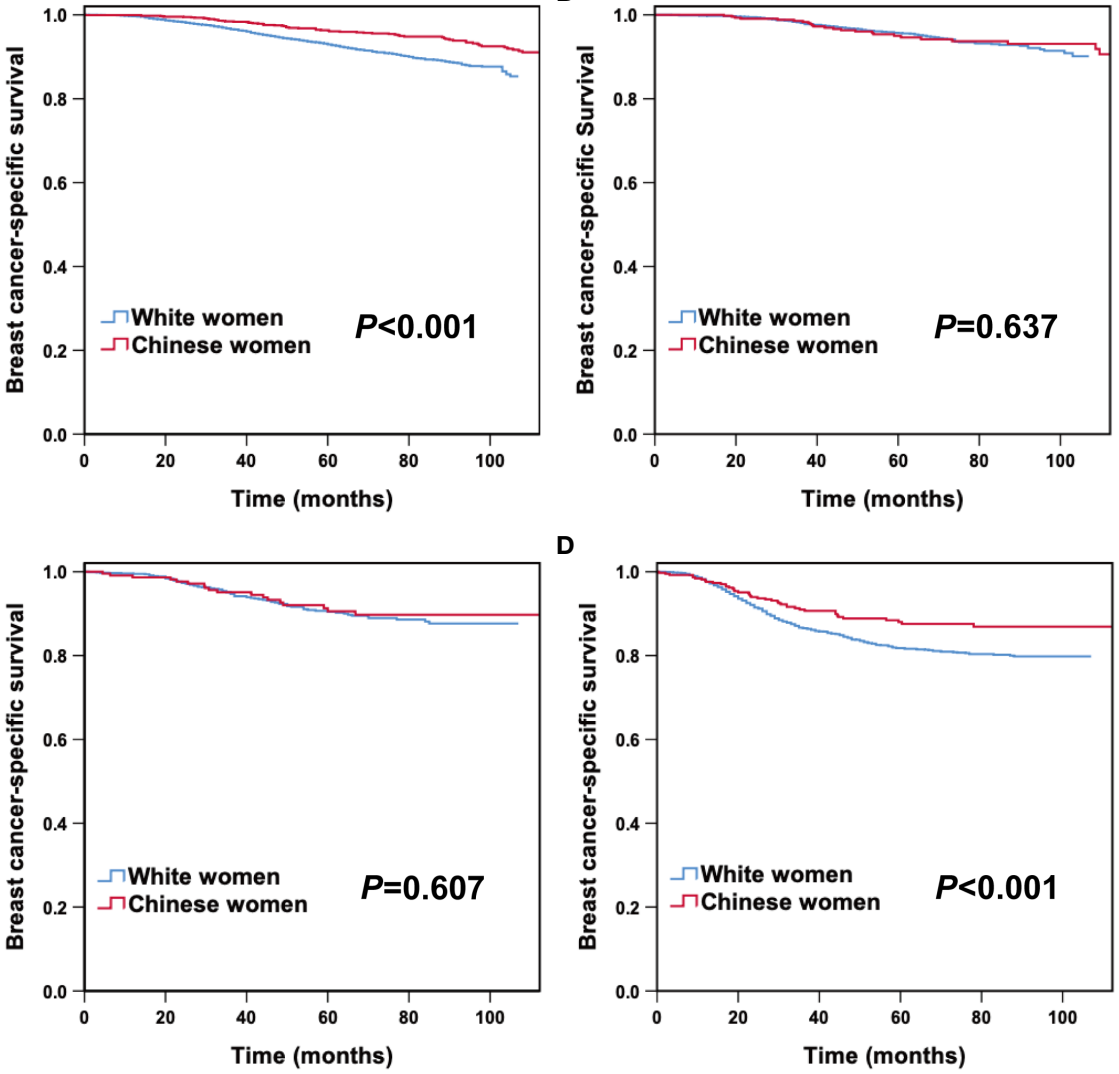
Breast cancer-specific survival between young Chinese and White breast cancer patients according to molecular subtypes. HoR (+)/HER2 (-) **(A)**, HoR (+)/HER2 (+) **(B)**, HER2 enriched **(C)** and triple-negative subtypes **(D)**.

After stratification by molecular subtype, we further analyzed the correlation between race/ethnicity and BCSS. Kaplan–Meier curves revealed that Chinese women had a superior 5-year BCSS in HoR (+)/HER2 (-) (96.3% vs 92.9%, *P*< 0.001), and triple-negative subtypes (88.0% vs 81.7%, *P*= 0.006) than White American women, while there were no survival differences between races/ethnicities in the HoR (+)/HER2 (+) and HER2 enriched subtypes (**Figures 2A–D**). As listed in **Table 4**, the HRs for BCSS were compared between young Chinese women and White American women in different molecular subtypes. In the cohort of patients with HoR (+)/HER2 (-) tumors, young Chinese women exhibited a lower risk of breast cancer-related death (HR: 0.373, 95% CI 0.275-0.506; *P*< 0.001) than White American women. Similarly, among triple-negative patients, young Chinese patients had better BCSS than White American patients (HR: 0.643, 95% CI 0.445-0.928; *P*= 0.018). However, for women with HoR (+)/HER2 (+) and HER2 enriched disease, race/ethnicity did not have a significant association with BCSS after adjustment for other relevant factors (*P*= 0.359 and *P*= 0.542, respectively) (**Table 4**).

**Table 4:** HRs for breast cancer-specific survival of race/ethnicity according to molecular subtype.

Variables	No. of Breast Cancer	Breast Cancer Deaths, No (%)	HR^a^	95% CI^a^	P value^a^
HoR (+)/HER2 (-)					
White American	10,508	600 (5.75)	1 (reference)	1	N/A
Chinese	1,389	70 (5.0)	0.373	0.275-0.506	< 0.001
HoR (+)/HER2 (+)					
White American	3,325	120 (3.6)	1	1	N/A
Chinese	460	24 (5.2)	0.795	0.487-1.298	0.359
HER2 enriched					
White American	1209	83 (6.9)	1	1	N/A
Chinese	226	18 (8.0)	0.819	0.430-1.557	0.542
Triple-negative					
White American	3358	449 (13.4)	1	1	N/A
Chinese	384	42 (10.9)	0.643	0.445-0.928	0.018

HR, hazard ratio; CI, confidence interval; HoR, hormone receptor; HER2, epidermal growth factor receptor 2.

a Adjusted for tumor grade, clinical stage, surgery, chemotherapy, and radiotherapy.

### Annualized hazard curve of breast cancer-related death by race/ethnicity

**Figure 3:**
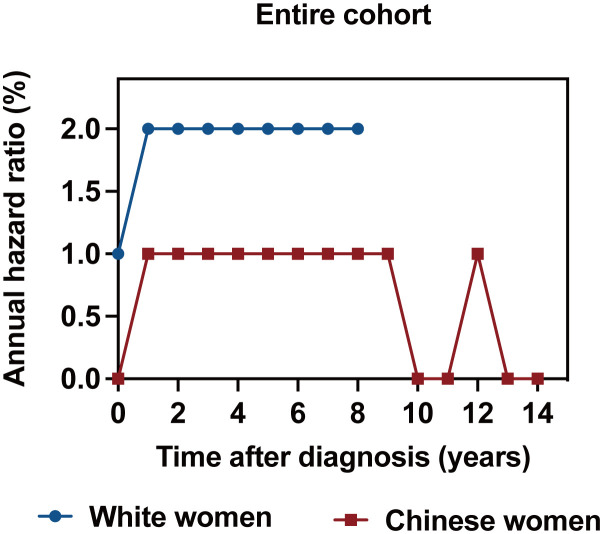
Annualized hazard curve of breast cancer-specific survival between young White women and Chinese women.

**Figure 4:**
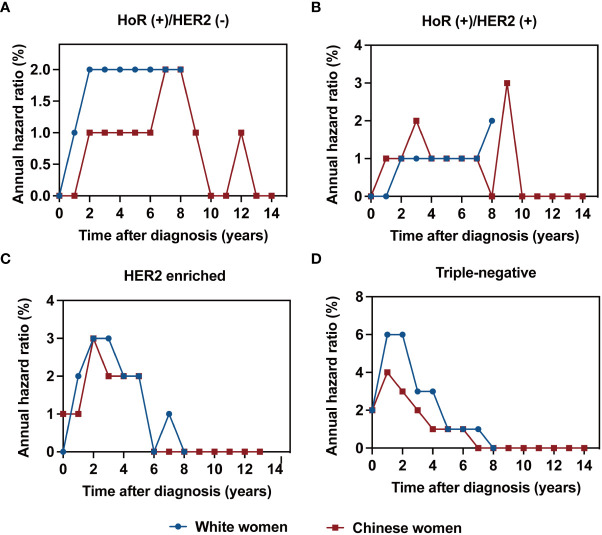
Annualized hazard curve of breast cancer-specific survival between young White women and Chinese women for HoR (+)/HER2 (-) **(A)**, HoR (+)/HER2 (+) **(B)**, HER2 enriched **(C)** and triple-negative subtypes **(D)**.

The annualized hazard trend of breast cancer-related death in the different races/ethnicities is presented in **Figures 3**, **4**. The annual breast cancer related-death risk rate of young White women was higher than that of young Chinese women within the first 8 years after diagnosis and did not reach a peak with a follow-up of 107 months (**Figure 3**). Then, the annualized hazard trends in the different races/ethnicities were further stratified by molecular subtype (**Figure 4**). Specifically, we found that in the HoR (+)/HER2 (-) molecular subtype, the hazard curve for breast cancer-related death of young White patients began to increase in the first year and then remained stable during the entire follow-up period (107 months). In the young Chinese population, the hazard curves began to rise in the second year, peaked in the seventh year, and subsequently declined before rising again in the twelfth year (**Figure 4A**). In the HoR (+)/HER2 (+) molecular subtype, the hazard curves of young White patients continued to increase over time and did not reach a peak within the follow-up period. In contrast, Chinese patients experienced two separate peaks in hazard rates, one in the third year (2.0%) and the other in the ninth year (3.0%), before declining (**Figure 4B**). Meanwhile, for the HER2 enriched molecular subtype, trends in the risk of breast cancer-related death were similar in White and Chinese patients during the first six years of follow-up, decreasing to zero in both groups in the sixth year and then remaining at zero percent in Chinese patients, while hazard rates of White patients reached 0.1% at the seventh year (**Figure 4C**). In the triple-negative molecular subtype, young Chinese patients still had a similar curve to young White patients, but the hazard rates in young Chinese patients were generally lower than in young White patients and dropped to zero in the seventh year, one year earlier than that of White patients (**Figure 4D**).

## Discussion

Using the data from SEER and West China hospital of Sichuan University to obtain information on breast cancer patients of White American and Chinese women prior to the age of 40, this study explored the differences in prognostic prediction and annualized hazard function between young Chinese and White American breast cancer patients across various molecular subtypes. Our findings demonstrated that although molecular subtype was a significant predictor in both young Chinese and White patients, different trends were observed in two cohorts when exploring the relationship between BCSS and molecular subtypes. Furthermore, the study revealed that young Chinese women had superior BCSS in HoR (+)/HER2 (-) and triple-negative breast cancers than young White American women. Finally, significant divergences were observed in the trends of the annual hazard function between the two racial/ethnic groups, particularly in the HoR (+)/HER2 (-) and HoR (+)/HER2 (+) subtypes. Overall, this study enhances our understanding of the biological characteristics of young breast cancer of diverse racial/ethnic backgrounds.

Young breast cancer patients have long been associated with unfavorable clinical outcomes, and recent research suggested that the correlation between age and prognosis varied by molecular subtypes (30, 31). Similar to prior studies (8, 22, 29), our research indicated that molecular subtype classification had an impact on survival disparities among younger breast cancer individuals. Our findings revealed that young women with triple-negative disease had the lowest BCSS rates regardless of race/ethnicity. Importantly, we found that in young White patients, the HoR (+)/HER2 (+) molecular subtype had the best prognosis, even better than the HoR (+)/HER2 (-) molecular subtype. This result could be related to the method of molecular subtype classification we employed. Due to the unavailability of Ki67 status in the SEER database, we classified molecular subtypes according to the status of HoRs and HER2, which could have resulted in some HER2-negative luminal B breast cancer patients being classified as HoR (+)/HER2 (-) (19, 28, 29). Another possible explanation for this result may be that younger women with luminal A breast cancer had a higher incidence of endocrine resistance, leading to a poor prognosis (25, 32). However, we observed that the HoR (+)/HER2 (-) subtype had a similar prognosis to the HoR (+)/HER2 (+) subtype in young Chinese breast cancer patients. This suggested that heterogeneity existed even within the same molecular subtype among young breast cancer patients of Chinese and White American ethnicity.

Racial/ethnic variation in BCSS has been well confirmed (33, 34). Several studies have explored racial/ethnic differences in breast cancer by incorporating molecular subtypes, but most of these studies have focused on disparities in survival among Black and White women, while less attention has been given to other races/ethnicities, including Chinese women (19, 26, 35). Using the National Comprehensive Cancer Network (NCCN) Breast Cancer Outcomes Database, a large study examined racial/ethnic disparities in BCSS according to subtype (22). The study included 17,268 women across the United States from different racial/ethnic backgrounds, including non-Hispanic White (White), non-Hispanic Black (Black) and non-Hispanic Asian or Pacific Islander (Asian). Their results showed that compared to White women, Asian women were at lower risk of breast cancer-related death for all molecular subtypes (HR, 0.56; 95% CI, 0.37- 0.85). However, when examining survival differences among different races/ethnicities within each molecular subtype, the statistical power of Asians in the investigation was constrained by the small number of inclusions (n= 533) and the low number of deaths (n= 24). Furthermore, because the distribution of the NCCN subtype was similar to that observed in population-based registries (36), the extent of racial/ethnic disparities in other cancer care outside of comprehensive cancer centers might be greater than what these findings indicated. Another study conducted by Caggiano et al. found that the relationship between age and breast cancer subtype may differ by race/ethnicity after adjustment for disease characteristics (23). Therefore, it is necessary to stratify age to further explore the differences in survival between different races/ethnicities according to molecular subtypes. As the incidence of breast cancer continues to increase among young Chinese women, it is crucial to recognize the survival variations between young Chinese women with other races/ethnicities across breast cancer subtypes. Our study included 2,459 young Chinese women from the SEER database and Chinese BCIMS database, which better reflected the basic situation of breast cancer in Chinese women in the real world. We observed that young White women had lower BCSS in the HoR (+)/HER2 (-) and triple-negative subtypes than young Chinese women, and even after considering tumor characteristics and treatment factors, survival differences persisted. The reason for the difference might be inherent genetic susceptibility. Limited evidence supports that there are significant differences in genomic mutations between Chinese women and White women with breast cancer. Studies have demonstrated an association between mutations in BRCA1 and triple-negative breast cancer (37), with variations in the spectrum of BRCA1 mutations observed between Chinese and White women, which may partially explain the survival disparities in triple-negative breast cancer among the two groups (38, 39). In addition, a study by Ren et al. reported that the incidence of TP53 and AKT1 mutations was higher among Chinese women (40). However, germline mutations in high and moderate penetrance susceptibility genes such as BRCA1/2 and TP53 occur at a lower frequency in breast cancer. With high frequencies in the population, common genetic variants associated with breast cancer risk, also exist distinctions between Asian and European populations (41, 42). A large-scale meta-analysis has revealed significant heterogeneity in breast cancer susceptibility loci, such as 5q11.2, 9p21.3, 12q24.21, 16q12.1, and 21q21.1, observed in Asian and European populations (42). While these findings may provide some insight into the observed disparities in BCSS between Chinese and White breast cancer patients, it is important to note that no identified genetic markers have been found to predict disparities in survival outcomes among patients of different races/ethnicities. Further study is required to comprehend the genetic variations among different races/ethnicities and their contribution to survival disparities among molecular subtypes.

In addition to extrinsic genetic factors, racial and ethnic differences are also influenced by other factors like demographic characteristics, treatment modalities and socioeconomic status. Studies have disclosed demographic differences between Chinese and White breast cancer patients, with Chinese patients displaying a younger age distribution and a greater proportion of married individuals (34, 43). Despite adjusting for these factors, disparities in survival between Chinese and White women persisted (34). Consistent with previous research, our findings revealed that Chinese women preferred to receive chemotherapy. In our study, Chinese women had a greater proportion of HER2-positive tumors, which may have increased their likelihood of receiving chemotherapy. However, after stratification by molecular subtype, it can be also observed that Chinese women had a higher proportion of chemotherapy compared to White American women across all molecular subtypes. Additionally, White American patients have more options for early diagnosis and adequate treatment than Chinese patients due to the economic development gap between the two countries (23). Nevertheless, even with better healthcare opportunities for White patients, the discrepancies in BCSS rates between Chinese and White women have not been eliminated (34). Unfortunately, the databases analyzed in this study did not comprehensively include the demographic and socioeconomic factors. It is indeed essential for future research to incorporate these important variables into consideration.

Recently, by comparing the annual mortality rates of breast cancer patients from the SEER database between American and Chinese women, a study found that White women exhibited a higher annual mortality rate than Chinese women during the 9 years after a breast cancer diagnosis, whereas Chinese women experienced a higher annual mortality rate than White women after the nine-year mark (34). However, this study did not stratify by age and molecular subtype. Similar to that study, our study revealed that young White women had a higher annual risk of breast cancer-related mortality than young Chinese women during the first 8 years following diagnosis. Due to the shorter follow-up time for White women, we did not observe the annual death risk rate of young Chinese women exceeding that of young White women. On the other hand, the results stratified by molecular subtype showed that the trends of the annual hazard function were significantly different between the two racial/ethnic groups in the HoR (+)/HER2 (-) and HoR (+)/HER2 (+) subtypes. Moreover, although the annual breast cancer death risk curves of young Chinese and White American patients with triple-negative breast cancer had similar trends, the peak value for White women was higher and lasted longer. Therefore, further exploration is needed to understand the reasons for disparities in survival and annual hazard function between young White women and Chinese women in different molecular subtypes and to guide the more personalized and precise treatment methods and management approaches.

There are still several limitations in our study. First, as a retrospective study, it inevitably has selection bias. There may also be potential confounding variables that could affect the results. Despite the possibility of variations in other factors among the study population, race/ethnicity remains a significant contributor to these disparities. Second, the data on Chinese women in this study came from two sources: the SEER database and the West China hospital of Sichuan University. Although it has been reported that no significant disparity in survival between Chinese breast cancer patients residing in the United States and those in China (34), it is necessary to note that those two cohorts might have variances in terms of lifestyle and environmental exposure. Third, this study may have potential time bias as a result of the inconsistent time intervals of diagnosed patients extracted from the two databases. To maximize the inclusion of individuals meeting the eligibility criteria, the year when each database initiated the recording of HER2 status was chosen as the respective starting point. Last, due to the lack of Ki67 status in the SEER database, this study was unable to accurately classify the luminal A and luminal B molecular subtypes according to current guidelines.

## Conclusion

In conclusion, our study revealed disparities in prognostic prediction and annualized hazard functions among young Chinese and White breast cancer patients with different molecular subtypes. In the era of precision oncology, it is necessary to ensure that future research has sufficient representativeness across all races/ethnicities. Evidence-based medicine is mostly derived from breast cancer populations in developed countries in Europe and America. Therefore, using White American breast cancer cases as a reference can clarify differences in breast cancer patients between China and the United States, which provides not only a reference to breast cancer in Chinese women but also a theoretical basis for developing and improving breast cancer treatment plans in China.

## Data availability statement

The datasets presented in this study can be found in online repositories. The names of the repository/repositories and accession number(s) can be found below: www.seer.cancer.gov.

## Ethics statement

This study received approval from the Ethics Committee of West China Hospital, Sichuan University (Approval number: 2020427). The patients/participants provided their written informed consent to participate in this study.

## Author contributions

LL contributed to conception and design of the study. YZ and JW: wrote the first draft of the manuscript. YZ, JW and XZ performed the statistical analysis, ZX, TY, SY, NX, and ZD organized the database. All authors contributed to manuscript revision, read, and approved the submitted version.
