# Supplementary material for: The disparities in prognostic prediction and annualized hazard function in different molecular subtypes between young Chinese and White American women with breast cancer
Source: Front Oncol. 2023 Jul 10;13:1199492. doi: 10.3389/fonc.2023.1199492 (PMC10364598; doi:10.3389/fonc.2023.1199492)
Supplement: Supplementary file 1 [file Table_1.pdf]

Table S1. The Receiving of Chemotherapy of Race/ethnicity According to Molecular Subtype.

**Variables**	**White women n= 18,400 (%)**	**Chinese women n= 2,459 (%)**	***P* value[a]**
**HoR (+)/HER2 (-)**			< 0.001
Chemotherapy	7,047 (67.1)	1,176 (84.7)	
No Chemotherapy	3,461 (32.9)	213 (15.3)	
**HoR (+)/HER2 (+)**			0.002
Chemotherapy	3,041 (91.5)	439 (95.4)	
No Chemotherapy	284 (8.5)	21 (4.6)	
**HER2 enriched**			0.002
Chemotherapy	1,085 (89.7)	217 (96.0)	
No Chemotherapy	124 (10.3)	9 (4.0)	
**Triple-negative**			0.047
Chemotherapy	3,107 (92.5)	366 (95.3)	
No Chemotherapy	251 (7.5)	18 (4.7)	

Abbreviations: hormone receptor: HoR; epidermal growth factor receptor 2: HER2.

aP value from chi-square test.
